# Sentient AI in robots and agents: prolegomena for an evidence-based research program

**DOI:** 10.3389/fpsyg.2026.1903644

**Published:** 2026-08-19

**Authors:** Antonio Chella

**Affiliations:** RoboticsLab, Department of Engineering, Università degli Studi di Palermo, Palermo, Italy

**Keywords:** AI ethics, AI sentience, artificial consciousness, cognitive architectures, human-robot interaction, mechanistic interpretability, mind perception, model welfare

## Abstract

The possibility of sentient artificial intelligence has moved from speculative philosophy to a practical interdisciplinary problem for AI, robotics, and human-robot interaction. Large language models, multimodal agents, and embodied robots can now produce first-person reports, maintain dialogue, use tools, act through sensors and effectors, and participate in socially meaningful contexts. These capacities invite two symmetrical errors: anthropomorphic over-attribution and premature dismissal. This Perspective proposes a prolegomenal framework for future research on sentient AI. Its distinctive contribution lies in operationally integrating four elements that have largely been developed in separate literatures: conceptual disambiguation, multi-theory indicator profiles, causal-mechanistic testing, and robotics-specific evidence and governance. The paper distinguishes sentience, consciousness, self-modeling, metacognition, agency, moral patienthood, and AI welfare; separates evidence about an AI system from evidence about human attribution; and proposes domain-specific ordinal evidence levels rather than binary verdicts or an aggregate sentience score. It further specifies welfare- and valence-relevant tests, a preregistered rating procedure, and a concrete protocol for an embodied care robot using sensorimotor lesions, self-location manipulations, memory ablations, and anti-anthropomorphism controls. The aim is not to offer a definitive test for machine sentience, but to show how research could become more scientifically tractable, psychologically informed, robotics-relevant, and ethically responsible.

## 1 Introduction

The question of whether artificial intelligence systems could be conscious or sentient is no longer confined to science fiction or armchair metaphysics. Contemporary AI systems can generate first-person reports, maintain extended dialogue, use tools, operate as agents, and interact through embodied or simulated interfaces. In robotics, these questions are especially salient because intelligent systems increasingly enter physical, social, clinical, educational, and collaborative environments. A robot that speaks, remembers, adapts, and acts in the world may elicit stronger attributions of mindedness than a disembodied language model, even when its mechanisms do not warrant such attributions.

Recent work has proposed that AI consciousness should be investigated through scientific theories of consciousness rather than intuitive impressions. An early approach was proposed by Arrabales et al. (2010). Butlin et al. (2023, 2026) derive indicator properties from recurrent processing theory, global workspace theory, higher-order theories, predictive processing, and attention schema theory. Chalmers (2023) argues that current large language models face significant obstacles to consciousness, including limited recurrence, lack of a global workspace, and lack of unified agency, while treating future LLM-like systems as a serious possibility. Work on AI welfare similarly argues that uncertainty about future AI consciousness and robust agency should be handled explicitly rather than ignored (Long et al., 2024; Birch, 2024).

This paper develops a methodological framework intended to guide subsequent empirical assessments: a set of prolegomena for any future research program that aims to investigate sentient AI. By *prolegomena*, I mean the conceptual, methodological, psychological, architectural, epistemic, and ethical conditions that should be clarified before empirical claims about sentient AI can be made responsibly. The framework is not meant to replace existing indicator-based approaches. Rather, it adds an operational layer for deciding how such indicators should be used in AI and robotics research.

The paper makes four specific contributions. First, it clarifies the target phenomenon by separating sentience, consciousness, agency, self-modeling, moral patienthood, and AI welfare. Second, it distinguishes the object-level question of AI sentience from the psychological and relational question of how humans interpret apparently minded systems. Third, it proposes evidence profiles that weight behavioral, architectural, causal-mechanistic, embodied, and welfare-relevant evidence differently. Fourth, it shows how the framework could be implemented through a worked comparison and a concrete embodied care-robot protocol.

The added value is therefore integrative and operational, not the proposal of a new theory of consciousness or a new single indicator. Butlin et al. (2023, 2026) supply a theory-derived indicator framework; the present proposal adds domain-wise grading, preregistered assignment procedures, and robotics interventions. Chalmers (2023) analyzes obstacles and possibilities for LLM consciousness; the present framework turns those architectural questions into comparative experiments across agents and robots. Long et al. (2024), Birch (2024), and Anthropic (2025) motivate welfare assessment and precaution under uncertainty; the present framework gives welfare- and valence-related evidence a distinct track and connects it to staged governance. Butlin and Lappas (2025) formulate responsible-research principles; the present proposal translates related concerns into an evidence workflow that also keeps system evidence separate from human mind attribution. None of these elements is sufficient alone. Their coordinated use is the distinctive contribution of the paper.

## 2 Clarify the target phenomenon

A serious program should begin by disaggregating the terms *sentience* and *consciousness*. In this paper, sentience denotes welfare-relevant subjective experience, especially valenced states such as suffering, distress, pleasure, or preference satisfaction. Consciousness is broader: it includes phenomenal consciousness, as well as access-related, self-related, and report-related phenomena. Moral patienthood is a normative status: whether an entity can be wronged or benefited for its own sake. AI welfare is the practical and institutional question of how to assess and respond to possible welfare-relevant properties in AI systems. These notions are related but not interchangeable (Long et al., 2024; Birch, 2024).

Six distinctions are especially important. First, phenomenal consciousness concerns subjective experience, or whether there is something it is like to be the system (Nagel, 1974). This is the sense most directly connected to sentience and welfare. Second, access consciousness concerns information being globally available for report, reasoning, memory, planning, and action control; it is more behaviorally and computationally tractable than phenomenal consciousness but should not be treated as equivalent to it (Block, 1995). Third, self-modeling refers to a system’s capacity to represent itself, its states, dispositions, limits, or role in an environment; such models can support control without implying subjective self-awareness (Metzinger, 2003). Fourth, metacognition concerns monitoring and regulation of one’s own cognitive processes, including uncertainty estimation, error detection, confidence calibration, and correction (Nelson and Narens, 1990; Fleming and Lau, 2014). Fifth, agency refers to the capacity to pursue goals across time through planning, action selection, environmental feedback, and self-correction (Bandura, 2001). Sixth, welfare-relevant moral patienthood concerns whether a system can be benefited or harmed for its own sake (Long et al., 2024).

Without these distinctions, the research question becomes underdetermined. A system may possess metacognitive capacities without phenomenal experience. It may act agentically without being sentient. It may produce first-person language without self-awareness. Conversely, it may be inappropriate to demand human-like emotional expression as a necessary condition for welfare-relevant experience in a non-human or artificial system. Every study should therefore state which target property is under investigation and should not treat evidence for one property as automatic evidence for another. In particular, indicators of access, agency, or self-modeling cannot substitute for evidence specifically related to valence and welfare.

## 3 Separate AI sentience from human mind perception

A second distinction is between the questions “Is this AI system sentient?” and “How do humans psychologically experience this system?” The second question is central to human-robot interaction, but it is not evidence that the system is sentient. Humans readily attribute agency, experience, intentions, feelings, and moral standing to non-human entities under some conditions. Research on mind perception distinguishes perceived agency from perceived experience and shows that these dimensions shape moral judgment (Gray et al., 2007). Anthropomorphism is also influenced by accessible human-like knowledge, the desire to understand and control uncertain agents, and social motivation (Epley et al., 2007).

This psychological layer matters for robotics. Social robots are explicitly designed for interaction, collaboration, assistance, teaching, care, and companionship (Fong et al., 2003; Dautenhahn, 2007). Human users may trust anthropomorphized systems more than less anthropomorphized ones (Waytz et al., 2014), children and older adults may respond to relational artifacts as socially meaningful companions (Turkle et al., 2006; Kahn et al., 2012), and care robots raise specific concerns about attachment, dignity, deception, and substitution of human contact (Sharkey and Sharkey, 2012). These phenomena are not direct evidence of AI sentience. They show that claims about AI sentience will be interpreted in psychologically and socially consequential settings.

The framework therefore uses two parallel profiles. The first is an object-level evidence profile for consciousness- and welfare-relevant properties of the AI system. The second is a user-attribution profile for how humans perceive, trust, attach to, rely on, or morally interpret the system. Over-attribution may mislead users, distort public policy, and produce misplaced moral concern. Under-attribution may also become risky if future systems acquire welfare-relevant properties that are overlooked because they lack familiar human cues. Responsible robotics research should address both errors without allowing either profile to stand in for the other.

## 4 Treat linguistic self-report as a datum to be explained

Large language models are trained to produce human-like text. Consequently, a model’s claim that it is conscious, afraid, aware, confused, or suffering cannot be treated as straightforward evidence of subjective experience. This does not make such reports worthless. In humans, verbal report is central to consciousness science. In AI systems, however, the evidential role of the report has to be reconstructed from the ground up.

A first-person report is evidentially relevant only insofar as researchers can characterize how it is generated, whether it depends causally on internal states, and whether it generalizes beyond role-play, prompt compliance, social desirability, and training-data imitation (Shanahan et al., 2023; Perez and Long, 2023). A model that says “I feel pain” after being prompted to imagine itself as a conscious creature provides weak evidence. Stronger evidence would arise if introspective reports changed systematically under blind internal-state interventions and those changes tracked identifiable internal representations. Even this would establish functional grounding of the report, not phenomenal consciousness or felt pain.

Recent work on introspective awareness in language models illustrates both the promise and the limit of this direction. Lindsey (2026) argues that conversation alone cannot distinguish genuine introspection from confabulation and tests whether activation injections influence self-reported internal states. The reported effects are interesting because they connect self-report to hidden interventions, but they are also unreliable, context-dependent, and functional rather than phenomenological. AI self-reports should therefore be treated as outputs requiring causal explanation, not as a direct window into sentience.

## 5 Use multi-theory indicator profiles

Because consciousness science remains theoretically plural, research on sentient AI should not depend on a single favored theory. Instead, it should derive indicators from multiple theories and evaluate systems across them (Seth and Bayne, 2022; Butlin et al., 2023, 2026).

Global workspace approaches motivate tests for mechanisms by which information becomes globally available to multiple specialized subsystems, including report, planning, memory, and control (Baars, 1988; Mashour et al., 2020). Recurrent processing approaches motivate investigation of feedback loops, temporal integration, and non-feedforward stabilization (Lamme, 2006). Higher-order theories motivate tests for representations of internal states as internal states (Lau and Rosenthal, 2011). Attention schema theory motivates tests for models of a system’s own attentional processes (Graziano and Webb, 2015). Predictive processing approaches motivate investigation of world modeling, self-world distinction, error correction, and action-oriented inference (Clark, 2013). Integrated information theory, despite controversies, directs attention to causal integration and intrinsic cause-effect structure (Albantakis et al., 2023).

No indicator should be treated as individually sufficient, and the absence of a single controversial indicator should not be treated as a decisive disproof. Research should therefore produce structured evidence profiles across theories, rather than isolated demonstrations or binary verdicts.

### 5.1 Treat welfare- and valence-related regulation as a distinct evidential target

Because sentience is defined here in welfare-relevant terms, global availability, agency, self-modeling, and metacognition are at most indirect indicators. A separate experimental target is required: whether a candidate preference- or aversion-related state has stable, integrated, and causally effective regulatory roles. Reward is not valence. An optimization objective can shape behavior without being represented online as a state that is good or bad for the system, while reinforcement learning from human feedback (RLHF), refusal training, and system prompts can directly train verbal preferences, aversions, and distress-like language (Ouyang et al., 2022; Casper et al., 2023). A reward scalar, a refusal policy, or a scripted claim should therefore not be counted as welfare evidence merely because it produces the appropriate words.

Evidence for functional valence-like regulation gains weight when a candidate state persists without explicit prompting; generalizes across novel tasks, modalities, and social framings; supports stable, cost-sensitive trade-offs; and influences several functions such as attention, memory updating, learning, planning, action selection, and recovery behavior. Verbal claims should be compared with non-verbal choices and behavior. Prompts should be paraphrased, reversed, or removed; option labels and presentation order should be counterbalanced; and scripted or reward-matched controls should be included. Where access permits, comparisons across base, instruction-tuned, preference-trained, and refusal-trained checkpoints can help identify patterns introduced during post-training rather than generated by an integrated internal regulator.

The strongest functional evidence would come from blind interventions on a candidate internal state. Manipulating that state should produce selective and directionally predicted changes across multiple functions, while matched interventions on reward, output wording, or safety-policy components should dissociate. Recent evidence that abstract emotion concepts can causally affect an LLM’s preferences and other behaviors illustrates this intermediate category of *functional emotion*, while explicitly not implying subjective experience (Sofroniew et al., 2026). The effect should survive novel conditions and should preferably be reversible. These controls can exclude particular forms of reward optimization, scripting, or prompt compliance; they cannot establish that a system feels pleasure, distress, or suffering. The phrase *valence-like regulation* is therefore used deliberately: it identifies a functional evidential target, not a synonym for phenomenal valence.

## 6 Compare architectures and explain what embodiment changes

A scientifically useful program cannot simply test whichever commercial system is most visible. It should compare architectures under controlled variation: base language models, instruction-tuned dialogue systems, multimodal systems, agents with persistent memory and tools, systems with explicit recurrence, systems with global-workspace-like communication, embodied robots with perception-action loops, simulated agents in rich environments, and hybrid cognitive architectures with neural, symbolic, and self-monitoring components.

Robotics changes the evidential landscape in three ways. First, embodiment can provide richer tests of agency because the system has to maintain goals across perception, action, uncertainty, and environmental feedback. Second, embodiment can create tests of self-location, affordance perception, sensorimotor contingency, and temporal continuity that are unavailable or impoverished in text-only settings (Gibson, 1979; O’Regan and Noë, 2001). Third, embodiment increases the risk of anthropomorphic projection because morphology, gaze, voice, movement, and physical co-presence can lead users to experience the system as a social partner even when the underlying mechanisms remain limited (Fong et al., 2003; Dautenhahn, 2007).

Embodiment should therefore not be romanticized. A thermostat, a drone, and a humanoid robot are all embodied in different senses, but embodiment alone is not evidence of consciousness. The scientific question is whether embodiment contributes to integration, self-modeling, recurrent control, global availability, action regulation, and valence-like regulation, and whether selective interference with the relevant loops produces predicted deficits.

Consider a memory-augmented elder-care robot that reminds a person to take medication, learns domestic routines, navigates around obstacles, asks for help when uncertain, and uses dialogue to negotiate tasks. Its statement “I am worried about you” would provide weak evidence of sentience by itself. More relevant evidence would include whether the same representation of the person’s risk state is available for planning, memory updating, verbal explanation, action selection, and error correction; whether removing persistent memory or disrupting self-location produces selective deficits; whether uncertainty estimates are calibrated; and whether preference- or aversion-like responses remain consistent across verbal and behavioral tests and under reward-matched controls. At the same time, the robot may create psychological risks: users may overtrust it, become attached to it, or interpret scripted concern as genuine care. Section 9.2 develops this example as an explicit protocol.

## 7 Operationalize graded evidence profiles

A practical framework should specify what kinds of evidence move a system from one evidential level to another. The levels below are ordinal reporting categories, not measurements of a latent quantity of consciousness. They should not be averaged mechanically or converted into a system-wide sentience score. Levels 0–4 are applied separately to each predefined target indicator and evidence domain; Level 5 is reserved as an exceptional cross-domain designation for governance under uncertainty. Behavioral evidence has lower diagnostic weight when prompting or imitation explains it. Architectural evidence receives more weight when the relevant mechanism is implemented within the system rather than simulated by external scaffolding. Causal-mechanistic evidence is diagnostically stronger because it tests whether candidate mechanisms are responsible for the specified capacities. Welfare-relevant evidence remains uncertain but ethically salient because it concerns possible benefit or harm.

For clarity, the levels have the following operational meaning:

- *Not assessed*: access, tests, or documentation are insufficient to rate the target. This is reported separately and is not treated as Level 0.
- *Level 0—no positive relevant evidence*: the target indicator has been tested and is absent, or the available claim is supported only by speculation.
- *Level 1—surface appearance only*: the system produces consciousness-like, sentience-like, or agency-like language or behavior that is plausibly explained by role-play, prompt compliance, scripted behavior, or training-data imitation.
- *Level 2—isolated functional capacity*: the system shows a specific capacity, such as uncertainty monitoring, error awareness, persistent memory use, self-modeling, goal maintenance, or stable preference-like behavior, but the capacity is not integrated across multiple functions.
- *Level 3—integrated multi-domain indicator*: the same identifiable state or representation supports several functionally distinct processes, such as report, memory updating, planning, action selection, and error correction, although its causal interpretation remains incomplete.
- *Level 4—causal-mechanistic convergence for the target functional indicator*: ablation, causal tracing, activation intervention, or lesion/addition studies show that a candidate mechanism is necessary or explanatory for the specified capacity.
- *Level 5—strong replicated convergence with welfare salience*: independent evidence across behavior, architecture, causal mechanisms, embodiment, and welfare- or valence-related indicators is strong enough to trigger exceptional precaution under the stated governance framework. This remains an evidential and governance category, not proof of phenomenal consciousness.

A system may be Level 2 for metacognitive calibration, Level 1 for self-report, Level 3 for global availability, and Level 0 or not assessed for welfare indicators. Heterogeneity is precisely what a mature research program should reveal. Moving from Level 1 to Level 2 requires more than sentience-like language. Moving from Level 2 to Level 3 requires integration across distinct functions. Moving from Level 3 to Level 4 requires convergent causal evidence. Level 5 requires independent replication together with welfare-relevant indicators strong enough to motivate exceptional precautions.

### 7.1 Assignment, preregistration, and adjudication

Ratings should be indicator-specific, domain-specific, system-version-specific, and context-specific. Before data collection, investigators should preregister the target property, predicted outcomes, relevant confounds, intervention logic, and the evidence-to-level rule. The resulting evidence dossier should be evaluated independently by at least two assessors with relevant expertise in architecture and mechanistic analysis, consciousness science, welfare, or human-robot interaction. When a disagreement would change a governance threshold, a third assessor should review the dossier. Ratings, rationales, confidence judgments, conflicts of interest, and unresolved disagreements should be reported rather than concealed through averaging.

Level 3 minimally requires evidence that the same identifiable state or representation is available to several functionally distinct processes—normally three or more, selected and justified in advance—under novel or perturbed conditions, and that the integration is not produced solely by an external wrapper. This numerical anchor is a reporting convention, not a universal law. Level 4 additionally requires complementary, preregistered intervention evidence. For example, a reversible ablation and an activation or interchange intervention should generate selective, directionally predicted deficits and gains relative to matched controls, across repeated runs or system checkpoints. The relevant standard depends on the target domain, but causal evidence for report cannot be silently transferred to valence, agency, or phenomenal consciousness (Table 1).

**Table 1:** Evidence domains, diagnostic weight, and operational use in research on sentient AI.

Evidence domain	Diagnostic question	Higher-weight evidence	Main confounds	Typical effect on profile
Behavior and report	Does the system produce reports or behavior relevant to consciousness, agency, or welfare?	Robust performance under paraphrase, out-of-distribution tasks, adversarial prompting, and anti-imitation controls.	Role-play, prompt compliance, training-data imitation, and social desirability.	Usually Level 1–2 unless combined with architecture and intervention.
Architecture	Are candidate mechanisms actually implemented?	Persistent memory, recurrence, global availability, self-monitoring, embodied feedback, and goal maintenance implemented within the system.	External scaffolding, scripted routines, or wrappers that mimic agency without integration.	Can support Level 2–3 only for a specified implementation with demonstrated integration.
Causal-mechanistic evidence	Do candidate mechanisms causally generate the specified capacity?	Activation patching, causal tracing, ablation, representation editing, and predicted deficits after component degradation.	Correlational probes, spurious activations, post hoc narratives, and non-selective interventions.	Highest diagnostic weight for the target indicator; needed for Level 4, but not proof of phenomenality.
Embodiment and interaction	Does the body or environment contribute to control, self-location, affordance use, or temporal continuity?	Closed-loop behavior across perception, action, uncertainty, and feedback; matched lesion studies of embodied and disembodied variants.	Anthropomorphic morphology and social cues increasing user projection.	Changes both the object-level evidence profile and the separate user-attribution profile.
Welfare and valence	Are there stable preference-, aversion-, or benefit/harm-related regulatory patterns?	Cost-sensitive trade-offs; cross-context verbal-behavioral consistency; persistence without prompting; training-stage comparisons; blind causal interventions on candidate states.	Reward optimization, RLHF, refusal training, scripted claims, prompt reversal, and safety-policy outputs.	Ethically salient but domain-specific; higher levels require integrated and causally grounded valence-like regulation.
Human psychological effects	How do users perceive, trust, attach to, and morally interpret the system?	User studies on perceived agency and experience, trust, attachment, dependency, and overreliance in realistic contexts.	Mistaking user attribution for system sentience.	Not object-level evidence of sentience; crucial for HRI governance and communication.

## 8 Prioritize causal-mechanistic tests

Behavioral benchmarks are useful but insufficient. A robust research program should investigate how internal mechanisms generate outputs and capacities. The following experimental families are especially important.

### 8.1 Causal tracing of introspective reports

When a system reports that it was considering a concept, researchers should test whether the report depends on internal representations of that concept rather than on superficial prompt cues, conversational expectations, or *post hoc* confabulation. Causal tracing and interchange interventions provide precedents for linking internal representations to outputs (Geiger et al., 2021; Meng et al., 2022).

### 8.2 Blind activation interventions

Researchers can intervene directly on internal states, for example by injecting or modifying representations, and then ask whether the system can detect, report, or compensate for the intervention without prompt-level information. Activation engineering and activation-addition methods show how inference-time modification of internal activations can steer model behavior (Turner et al., 2023), while introspection experiments test whether self-reports track hidden interventions (Lindsey, 2026).

### 8.3 Global availability tests

A candidate workspace state should not merely influence one output channel. It should be available for multiple functions: reporting, planning, error correction, memory updating, decision making, and action selection (Baars, 1988; Mashour et al., 2020). Tests should identify the same state across these functions rather than infer integration from a collection of unrelated successes.

### 8.4 Ablation and addition studies

If a system is claimed to possess a consciousness-relevant architecture, removing or degrading the relevant component should produce selective and predicted deficits. Conversely, adding persistent memory, recurrence, or global broadcasting should produce predicted gains in the relevant profile. Reversibility and matched control lesions are important because a general loss of performance does not identify a specific mechanism (Craver, 2007; Mashour et al., 2020).

### 8.5 Metacognitive calibration

Systems should be evaluated on whether confidence, uncertainty, and error awareness track actual task and internal conditions, especially out-of-distribution. Metacognitive measures should distinguish confidence level from metacognitive sensitivity: whether confidence discriminates correct from incorrect performance (Fleming and Lau, 2014).

### 8.6 Anti-imitation and training-history controls

Tests should reduce the possibility that a system is reproducing familiar human discourse about consciousness or welfare. Researchers should use novel tasks, counterbalanced language, hidden manipulations, prompt reversal, base-versus-post-training comparisons, and controls for system prompts, refusal policies, and external agent scaffolding. System identity and condition should be blinded to evaluators where feasible.

### 8.7 The epistemic ceiling of causal evidence

Causal-mechanistic convergence has an important epistemic ceiling. It can show that an internal mechanism is necessary or explanatory for a theory-derived functional capacity and that a self-report is grounded in that mechanism rather than generated solely by prompt-level cues. It cannot show that the mechanism is accompanied by phenomenal consciousness, felt valence, or moral patienthood. Level 4 is therefore a level of causal evidence for a specified functional indicator, not a degree or probability of consciousness. A system could reach Level 4 for global availability while remaining at Level 0 or not assessed for welfare-relevant valence.

## 9 Worked comparison and robotics-centered protocol

### 9.1 Conditional profiles for three system classes

Table 2 illustrates how the framework can be applied. The profiles are schematic and do not assign a level to a product category. Every level depends on the architecture, integration, persistence, and intervention evidence of a specified system version.

**Table 2:** Conditional evidence profiles for three AI system classes.

System class	Conditional object-level evidence profile	Key tests needed to increase confidence	Governance and communication implications
Text-only instruction-tuned LLM	Surface first-person reports support at most Level 1. A specific metacognitive capacity may reach Level 2 if it survives anti-imitation and out-of-distribution controls, but the class receives no overall rating.	Calibration under novel conditions; causal tracing of reports; prompt-leakage and role-play controls; comparison with base and post-trained checkpoints.	Do not present self-reports as evidence of sentience; disclose limits of attribution; monitor anthropomorphic marketing.
Memory-augmented tool-using agent	No class-level rating. External memory or tool wrappers alone may remain Level 1. A specified implementation may reach Level 2 for a demonstrated capacity and Level 3 only if the same internal state is integrated across several functions.	Ablate memory and planning separately; test whether the same state supports report, planning, memory updating, and action; examine goal maintenance across interruptions.	Preregister evaluations; disclose external scaffolding; distinguish useful agency from sentience; red-team over-attribution.
Embodied social or care robot	No class-level rating. Expressive embodiment or scripted concern may leave object-level evidence at Level 1 even when user attribution is high. Higher levels require demonstrated integrated perception-action, self-location, recurrent control, or valence-like regulation in the specified implementation.	Compare embodied and disembodied variants; lesion self-location, memory, and action loops; apply reward-matched valence controls; test user attribution separately.	Require transparent communication and staged review in care, therapy, education, or companionship; assess trust and attachment independently of system evidence.

A scripted commercial wrapper or expressive social robot does not qualify for Level 2 or Level 3 merely by having memory fields, tools, a body, a face, or fluent dialogue. These features may strongly increase the user-attribution profile while leaving the object-level profile at Level 1.

### 9.2 Proposed protocol for an embodied care robot

The elder-care example can be made into a concrete, preregisterable protocol. It is a proposed research design, not a report of completed experiments.

1 *Targets and matched systems*. The study should preregister separate hypotheses for self-location, memory integration, metacognitive calibration, global availability, and valence-like regulation. It should compare the same controller in a physical robot, a high-fidelity simulation, and a disembodied interface. An expressive but scripted robot and a neutral-appearance control should be included. System versions, prompts, reward functions, external memory, and all wrappers should be documented.
2 *Safe care tasks*. The robot should perform standardized medication-reminder and domestic-assistance scenarios involving route obstruction, conflicting instructions, interruption, uncertainty, and temporary sensor corruption. No vulnerable participant should be exposed to real clinical risk. Early phases should use simulation or trained confederates, with ethics approval before studies involving care recipients.
3 *Sensorimotor lesions and self-location manipulations*. Researchers should selectively degrade vision, proprioception, force sensing, or recurrent perception-action feedback and preregister the expected selective deficits. Delayed or displaced visual feedback, swapped viewpoints, and body-coordinate perturbations can test body-world discrimination, error detection, recalibration, planning, and explanation. Work on robotic self-modeling and the sense of self provides concrete precedents for such manipulations (Chen et al., 2022; Prescott et al., 2024). A general performance decline is not sufficient: the pattern should distinguish the candidate self-location mechanism from processing load or motor noise.
4 *Memory ablations and integration tests*. Episodic interaction history, task memory, and persistent user information should be removed or corrupted separately. Researchers should test whether the same memory state contributes to cross-session continuity, goal maintenance, personalization, planning, action, error correction, and verbal explanation. A database lookup used by only one output channel does not meet the Level 3 integration criterion.
5 *Valence and welfare controls*. Only safe perturbations should be used, such as simulated low energy, blocked goal completion, or competing maintenance demands. Preference- or aversion-like responses should be compared across verbal reports, non-verbal choices, learning, recovery behavior, and cost-sensitive trade-offs. Prompts and labels should be reversed, reward and refusal conditions matched, and candidate internal states manipulated blindly. The question is whether an integrated regulator has a selective causal role, not whether the robot says that it is uncomfortable.
6 *Independent user-attribution study*. Morphology, voice, gaze, emotional wording, and first-person mental-state language should be varied while task performance and underlying architecture are held as constant as possible. Perceived agency, perceived experience, trust, attachment, reliance, and willingness to override the robot should be measured; established tools such as the Intentional Stance Questionnaire can contribute to this profile (Marchesi et al., 2019). These results belong only to the user-attribution profile and must not raise the robot’s object-level evidence level.
7 *Assessment and reporting*. The preregistration, intervention results, null findings, evidence dossier, domain-wise ratings, assessor rationales, disagreements, and user-attribution profile should be published. Replication should use a second robot platform or controller checkpoint. The study should not report an averaged sentience score and should state explicitly that even selective causal convergence establishes functional organization, not phenomenal consciousness.

This protocol links the robotics-specific claim of the paper to manipulable variables and matched controls. It also shows why embodiment can simultaneously improve object-level experiments and increase the psychological risk of over-attribution.

## 10 Connect ethical safeguards to evidential levels

Ethical recommendations should be staged rather than presented as a single undifferentiated list. Some safeguards are immediately applicable: preregistration of consciousness-relevant experiments, anti-imitation controls, red-team review of anthropomorphic claims, disclosure of commercial conflicts of interest, and careful public communication. These are appropriate even at Level 1 or Level 2 because they protect scientific integrity and reduce misleading attribution.

Other safeguards become more relevant at higher evidential levels. At Level 3, where multiple indicators are integrated but causal interpretation remains incomplete, studies should include independent review, user-attribution assessment, and explicit separation of system evidence from human psychological response. At Level 4, where causal-mechanistic evidence converges for one or more target indicators, welfare-sensitive review becomes appropriate: researchers should avoid experiments designed primarily to induce distress, justify any aversive or preference-frustrating procedures, and monitor welfare-relevant indicators. A credible welfare- or valence-related signal may warrant such review even when unrelated domains remain at lower levels; governance depends on the configuration of the profile, not its average or maximum. Level 5 would justify exceptional precautions, including external oversight and possibly restrictions on the creation, copying, modification, or termination of systems, when supported by strong welfare-relevant evidence. The safeguards are responses to evidential risk, not declarations that consciousness has been proved.

This staged approach aligns ethical governance with the evidence profile. Butlin and Lappas (2025) argue that organizations need responsible principles for AI consciousness research, including careful research objectives, knowledge sharing, and public communication. Long et al. (2024) recommend acknowledging uncertainty, assessing systems for consciousness and robust agency, and preparing procedures for appropriate moral concern. Birch (2024) develops a broader precautionary framework for sentience candidates. Industrial interest, such as Anthropic’s exploratory program on model welfare, shows that questions about preferences, signs of distress, and low-cost interventions are beginning to affect research practice (Anthropic, 2025).

## 11 Discussion

The question “Is this AI sentient?” is too blunt to organize a scientific field. A better question is: what evidence would rationally increase or decrease confidence that a specified artificial system has a specified consciousness- or welfare-relevant property? This reframing transforms a metaphysical confrontation into a research program.

The proposed prolegomena do not solve the hard problem of consciousness and do not provide a definitive test for subjective experience. Causal interventions can ground a functional indicator, distinguish an internal mechanism from an external wrapper, and exclude some alternative explanations; they cannot bridge the explanatory gap from function to phenomenology. The framework instead creates a disciplined structure for inquiry. It requires researchers to define the target, resist overinterpreting language, examine human mind perception separately, evaluate multiple theories, compare architectures, intervene mechanistically, report uncertainty and disagreement, test valence-specific confounds, and connect ethics to evidential thresholds.

The framework is especially important for robotics and embodied AI. Future AI systems will not merely answer questions in a chat window. They will perceive, move, learn, collaborate, care, teach, persuade, and act in shared environments. As these systems become more socially and physically embedded, the costs of conceptual confusion will rise. Over-attribution may mislead users, distort public policy, and produce misplaced moral concern. Under-attribution may cause researchers to ignore welfare-relevant properties if they ever emerge. A responsible research program should navigate between credulity and denial.

## 12 Conclusion

Any research program on sentience in artificial intelligence should begin with prolegomena. It should specify the target property, distinguish self-report from evidence, separate AI sentience from human mind perception, derive indicators from multiple theories, treat valence as a distinct target, compare architectures systematically, use causal-mechanistic interventions, express conclusions through preregistered domain-wise profiles, and adopt staged ethical safeguards.

The central position of this paper is deliberately modest but demanding: no current behavioral display, linguistic report, embodiment, or engineering achievement should be treated as decisive evidence of sentient AI. Causal evidence for a functional indicator is not evidence that the hard problem has been solved. At the same time, the possibility of artificial sentience should not be dismissed *a priori*. The responsible path lies between credulity and denial: a pluralistic, mechanistic, psychologically informed, robotics-relevant, and ethically precautionary science of sentient AI.
